# Competing for Attention in Social Media under Information Overload Conditions

**DOI:** 10.1371/journal.pone.0126090

**Published:** 2015-07-10

**Authors:** Ling Feng, Yanqing Hu, Baowen Li, H. Eugene Stanley, Shlomo Havlin, Lidia A. Braunstein

**Affiliations:** 1 Department of Physics and Centre for Computational Science and Engineering, National University of Singapore, 117542, Singapore; 2 Complex Systems Programme, Institute of High Performance Computing, Agency for Science Technology and Research, 138632, Singapore; 3 School of Information Science and Technology, Sun Yat-sen University, Guangzhou 510006, China; 4 School of Mathematics, Southwest Jiaotong University, Chengdu 610031, China; 5 Center for Phononics and Thermal Energy Science, School of Physics Science and Engineering, Tongji University, 200092, Shanghai, China; 6 Center for Polymer Studies and Department of Physics, Boston University, Boston, MA 02215, United States of America; 7 Department of Physics, Bar-Ilan University, 52900 Ramat-Gan, Israel; 8 Instituto de Investigaciones Fisicas de Mar del Plata (IFIMAR), Universidad Nacional de Mar del Plata-CONICET, Funes 3350, (7600) Mar del Plata, Argentina; National Scientific and Technical Research Council (CONICET)., ARGENTINA

## Abstract

Modern social media are becoming overloaded with information because of the rapidly-expanding number of information feeds. We analyze the user-generated content in Sina Weibo, and find evidence that the spread of popular messages often follow a mechanism that differs from the spread of disease, in contrast to common belief. In this mechanism, an individual with more friends needs more repeated exposures to spread further the information. Moreover, our data suggest that for certain messages the chance of an individual to share the message is proportional to the fraction of its neighbours who shared it with him/her, which is a result of competition for attention. We model this process using a fractional susceptible infected recovered (FSIR) model, where the infection probability of a node is proportional to its fraction of infected neighbors. Our findings have dramatic implications for information contagion. For example, using the FSIR model we find that real-world social networks have a finite epidemic threshold in contrast to the zero threshold in disease epidemic models. This means that when individuals are overloaded with excess information feeds, the information either reaches out the population if it is above the critical epidemic threshold, or it would never be well received.

## Introduction

Because of the expanding size of such online social networks (OSNs) as Facebook and Twitter, modern media carry an enormous amount of user generated content. As their impact on society is increasing, much interest is now being focused on the spreading mechanism in social networks. In order to understand the mechanisms underlying information diffusion, many studies involve analyzing large amounts of empirical data [1–7], and others formulate predictions of how popular a particular piece of information will become [8, 9]. The susceptible infected recovered (SIR) model [10] of disease epidemics is frequently used to model the spread of information.

Although disease, opinion, and information spreading all share significant similarities, fundamental differences remain. In the spread of disease [11–15], every person coming in contact with an infected individual has the same probability of being infected, and the infected individual continues to infect others until it no longer has the disease. In contrast, an individual with many friends may not share a held opinion if only a few of the friends agree with the opinion. Indeed, in many opinion models [16–29] individuals strongly tend to conform to the majority opinion of their friends. The spreading of information on OSNs is similar to that in epidemic and binary-choice opinion models, but the detailed mechanisms can differ. Studies [30, 31] have shown that the epidemic models do not reproduces certain empirical statistics observed in information spreading, and modifications on ‘inactive’ and ‘ignorant’ behaviours could significantly improve model results, and the influence of super-spreaders are present to ensure the matching statistics between models and empirical data. Social experiments have found that individuals often adopt new social behaviors when they are strongly influenced by repeated signals from friends [32], and extensive empirical study of Facebook found that the predominant component of Internet content spreading is the influence of “weak” links, e.g., the viewing of content generated by individuals with whom the viewer has had no interaction. This suggests that these weak links play a much more important role in information diffusion in OSNs than in face-to-face social networks where there is social interaction [33]. This same study [33] and others [34] also found that most of the spreading of online information occurs within the first day of its posting, indicating a short lifetime and a decaying rate of diffusion similar to that in the spread of disease. In addition to the social reinforcement behaviors discovered in Ref. [32], Ref. [34] also found that highly connected nodes (individuals with many friends) are less likely to spread (pass on) incoming information.

Although most empirical analyses focus on large data sets and use average statistics as a reference when determining universal spreading mechanisms, there is evidence [35] that spreading modes differ as information types differ and that they are also influenced by the number of linked “friends” a user has. Here we examine a set of popular messages on the Chinese microblog site Sina Weibo to determine the number of repeated message “shares” an individual needs to receive before they in turn share the message. We find that much of the viral spreading of popular messages follows a fractional SIR model, i.e., an individual with a large number of friends needs more repeated signals before sharing a message than one with a few number of friends. The analysis also suggests that the probability that an individual will share a message is proportional to the fraction of its friends that have shared it with him or her. This mechanism leads to a phase transition behavior in the spreading, i.e., messages with attractiveness below a critical threshold will not spread to a significant fraction of the population of the OSN, and those above the critical threshold will.

## Empirical Motivation

### Data Source

**Fig 1:**
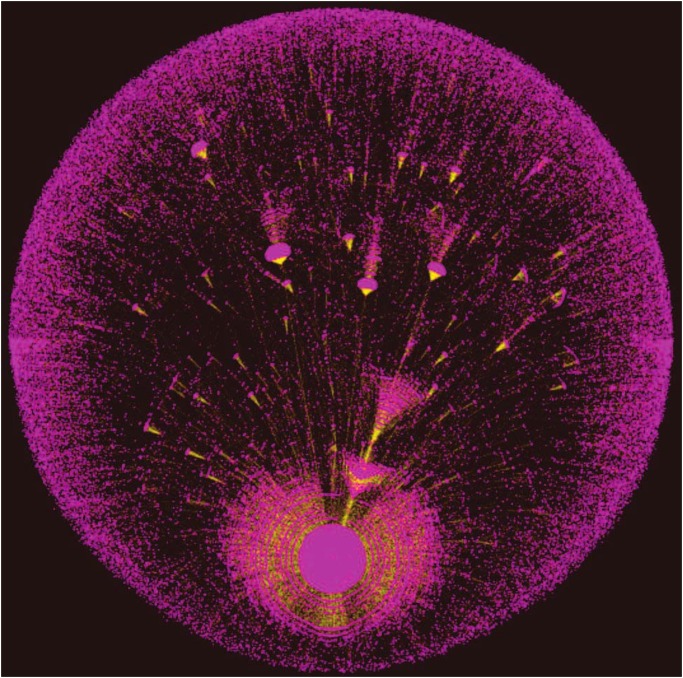
Graphical representation of a diffusion tree on Sina Weibo. This is a graphic representation for one of the popular messages on Weibo, and the branching process can be seen. It has been shared more than 190,000 times. The purple dots represent users who have shared the message, and green lines represent the paths through which the messages are spread. If a user A shared the message from user B, a green line is drawn between both. The clusters of purple dots corresponds to a user with a large number of followers who have shared the message from this user.

We obtain our data from the micro-blog site Chinese Sina Weibo (www.weibo.com), one of most popular social media channels in China. It is similar to Twitter and by the end of 2012 had more than 40,000,000 active users [36]. A user can view messages that other users post but cannot send messages to them unless the other users elect to “follow” this user. The messages are limited to 140 Chinese characters. The number of people a user with a free account can follow is limited to 2000. (A tiny number of users elect to have the paid account option that allows a higher limit.) Fig 1 shows the transmission pattern for one of the popular messages on Weibo. The purple dots are users who have shared the message, and the green lines are the paths through which the message has spread. The spreading is a branching process that originates from a first node near the bottom of the figure.

In many empirical studies of information diffusion and user behavior in social media, aggregate statistics have been derived using a large amount of information content [7, 37]. Although this approach has the advantage of having large data sets and reliable statistics, it ignores the heterogeneity of information content and its effect on any findings.

In order to examine how message transmission dynamics vary as the type of message varies, we select the most popular 286 messages that were shared at least 40,000 times in December 2012 (data available in S1 File). Because we focus solely on these popular messages, we avoid the need to identify and filter spam [38]. For each message, we obtain the diffusion process by identifying every node (every user account) that has shared the message and recording the time of each sharing. For each node in this diffusion tree, we get the number of times it receives the same message from friends before it in turn shares the message. We do this by combining the network structure data with the sharing-time data.

### Empirical Observations

We first define three quantities:

- The number of users *N*_*i*_ sharing message *i*, which in epidemic spreading terms corresponds to the total number of individuals who have been infected and have recovered.
- The fraction of users sharing the same message as *R* = *N*_*i*_/*N*, where *N* is the total number of active Weibo users.
- The total number of followees of user *j* (the total number of users being followed by *j*) who share share a message with *j* before *j* shares the message is defined as $k_{j}^{-}$. In the epidemic model this corresponds to the number of infected neighbors of node *j* before *j* is infected. The total number of users that user *j* follows is defined as *k*_*j*_. For each message, we denote the average of $k_{j}^{-}$ of all the users sharing this same message as ⟨*k*_⟩.

**Fig 2:**
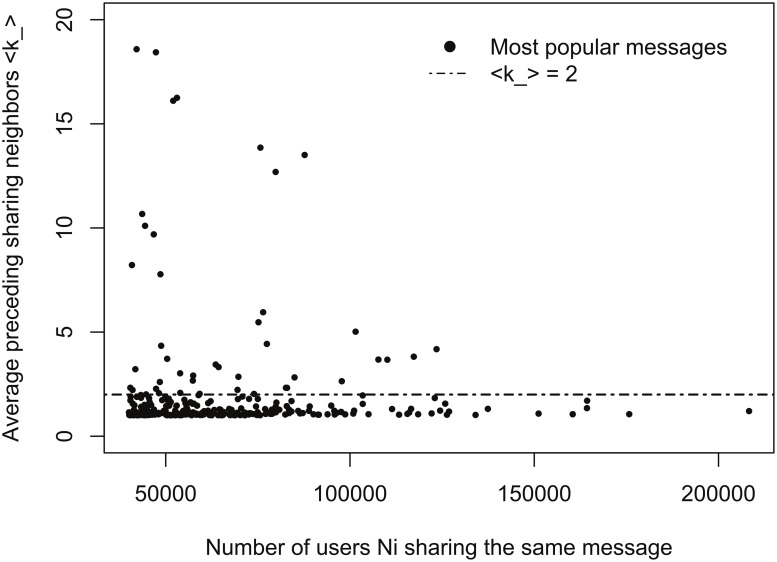
Plot of ⟨*k*_⟩ for each of the the most popular messages as a function of *N*_*i*_. Messages are the most popular ones from Sina Weibo in December 2012. Majority of the most popular messages have average preceding sharing neighbors smaller than 2, which contradicts the SIR model. This hints that the mechanism might be different from SIR models for disease spreading for scale-free network of OSN type.

Fig 2 shows that in the majority of these messages have an average value ⟨*k*_⟩ < 2. This is in contrast to the SIR model of scale-free networks in which, as we examine in more detail below, ⟨*k*_⟩ > 2.

Thus the hypothesis of our new model is that the spread of information in an OSN differs from the spread of a disease during an epidemic. In an OSN users pay only limited attention to incoming information and as the number of their contacts increase this attention to each contact decreases further. In the following section we test this hypothesis by comparing the outcome from our model with real-world data.

## The Fractional SIR (FSIR) Model

In our fractional SIR (FSIR) model we assume that, as the number of friends a user has increases, the number of enforcements from these friends of a particular message required before the user passes the message on (“spreads the infection further”) also increases. Because the total amount of attention a user can pay to the OSN is limited, we hypothesize that the amount of influence from each neighbor is inversely proportional to the total number of contacts (followees) the user has.

Evidence exists [39] that despite the large amount of information a node with high connectivity *k* can *receive*, the amount of information it *shares* is not significantly greater than the amount of information shared by a node with low connectivity *k*. Thus the total amount of attention a node can pay to the total information received from all of its neighbors is limited, irrespective of the node’s connectivity (there is a cognition limit). If all nodes thus have approximately the same cognition limit, an increasing overload of information will cause the higher-degree nodes to pay a decreasing amount of attention to information received from each of its contacts.

Our model assumes a network of *N* nodes, each representing a user account on the OSN in which the number of contacts *k* of a node is given by a degree distribution *P*(*k*). In the real-world Weibo network the links are directed because the follower of a user account can see its posts but the user account cannot see the posts of its followers. For simplicity we use undirected networks in our analysis. Reference [40] shows that on the microblog site Twitter the users sharing the most messages are also the ones receiving the most messages despite the fact that the number of their followers differ from their number of followees. Hence an undirected network is a good approximation of message flow in a social network. The spreading mechanism in our model is as follows:

1. A node has information (is infected) at step *t* = 0. At a subsequent time *t*, for each node *i* that has not shared the information (has not been infected but is susceptible) but has friends that have (have been infected), the probability that *i* will subsequently share the information (the infection) from each infected (sharing) friend (followee) is γ/*k*_*i*_, where *k*_*i*_ is the degree of node *i*.
2. τ time steps after sharing (infection), an infected individual recovers and cannot be infected again (is no longer visible to its neighbors on the Internet). Hence the probability a susceptible node will be infected by a sharing friend is *T*_*k*_*j*__ = 1 − (1 − γ/*k*_*j*_)^τ^, where *k*_*j*_ is the degree of the susceptible node *j*.
3. At the final steady state nodes can no longer be infected, and all of the nodes in are in the recovery or susceptible state. The message is no longer being shared.

Here γ is the intrinsic attractiveness of the information, and τ is the visible duration on the feeds of a user’s followers.

Step one of our model differs from the corresponding step in the SIR model. In the SIR model, the probability of infection is γ (and is independent of the degree of the susceptible node), and the effective probability of infection is *T* = 1 − (1 − γ)^τ^ and is independent of *k*. In contrast, the infection probability in our FSIR model is determined by the individual node degree γ/*k*. Thus a node of degree *k* can be infected with probability *T*_*k*_ = 1 − (1 − γ/*k*)^τ^. In a real-world OSN, *k* ≫ γ, and thus *T*_*k*_ ≈ γτ/*k* = Γ/*k*, with Γ ≡ γτ. In the case of SIR, *T* ≈ Γ for any node of any degree *k*.

We perform FSIR and SIR simulations on a scale-free network of size *N* = 100,000 and degree distribution *P*(*k*) ∼ *k* ^−λ^, with λ = 2.5, which is the approximate empirical degree distribution of real-world OSNs. We fix the average degree at ⟨*k*⟩ = 50 to stay close to real-world OSNs. Since the popularity of the empirical data is typically less than 0.5% of the total population of users (200,000 shares out of 40,000,000 active users), i.e., *R* ≈ 0.5%, we use Γ values in our simulations that give similar *R* values, which corresponds to *N*_*i*_ ≈ 500. We run 5000 realizations for both the FSIR and the SIR model, and select out the realizations in which *N*_*i*_ > 200, which corresponds to the *R* values of the highly popular messages selected from real-world data.

**Fig 3:**
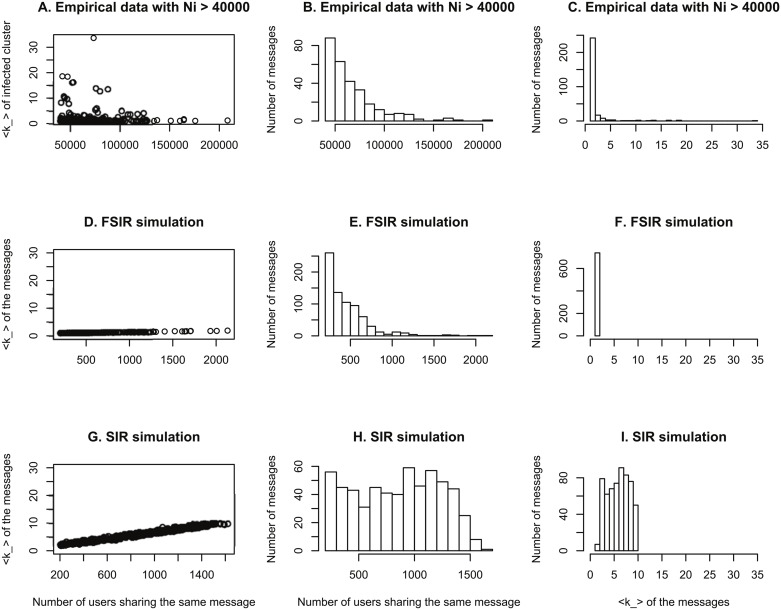
Comparison between empirical data and simulation results of FSIR and SIR models. In the simulation, the total number of nodes is *N* = 100,000. The degree distribution is chosen at *P*(*k*) ∼ *k* ^−2.5^, *k*_*min*_ = 20 and ⟨*k*⟩ ≈ 50. With these parameters this distribution is close to the empirical distribution of OSNs. The chosen parameter Γ = 0.7 for FSIR and 0.15 for SIR is such that the fraction of infected nodes *R* = *N*_*i*_/*N* ≈ 0.5%, is close to the empirical *R* values of the most popular messages. As we can see, for empirical and FSIR, ⟨*k*_⟩ values does not change significantly with *N*_*i*_ as seen in A and D. However, as seen in G, SIR shows a clear increase of ⟨*k*_⟩ as a function of *N*_*i*_. As seen in D and F, most of the real messages and FSIR result have ⟨*k*_⟩ < 2, meaning they are small outbreaks below epidemic threshold. But in I, we see that for SIR simulations, ⟨*k*_⟩ > 2. This means the messages are epidemics spreading rather than small outbreaks. In B and E, we show that both real data and FSIR have similar distributions of *N*_*i*_ values, with number of messages decreases significantly with increasing popularity *N*_*i*_. This is in contrast to SIR result in H. Hence through simulations, we show that FSIR captures the statistics of real data from popular messages, yet SIR does not.

Fig 3 shows the simulation results for FSIR and SIR models with Γ values that give outbreak fractions around 0.5%. In order to match the empirical *R* value of 0.005, we assume Γ = 0.7 for FSIR and Γ = 0.15 for SIR. Comparing A, D, and G in Fig 3, we see that FSIR produces ⟨*k*_⟩ values below 2 that do not change significantly with *N*_*i*_, which is similar to the empirical observation in A. In contrast, the range of ⟨*k*_⟩ values in the SIR model is mostly above 2 and the values increase with *N*_*i*_. Later we show that the FSIR has a phase transition at Γ_*c*_ ≈ 1. This suggests that the most popular messages will spread below a critical threshold, which is similar to the FSIR mechanism but not to the SIR. Comparing B, E, and H, we see that the FSIR model produces a distribution of *N*_*i*_ that decreases as *N*_*i*_ increases, which similar to the empirical distributions. In contrast, the distribution of the SIR model is uniform. Fig 3C, 3F and 3I show that the number of messages with values of ⟨*k*_⟩ < 2 in the empirical data clearly resemble those of the FSIR simulations, not the SIR simulations in which most of the *k*_ values are above 2. Thus the FSIR simulations capture the statistical properties of real data closely and the SIR simulations do not.

Our analysis thus strongly suggests that highly popular messages spread with a mechanism closely modeled by FSIR simulations but not conventional SIR simulations. This supports the hypothesis that a user with a larger number of neighbors will be proportionally less influenced by each of them, a clear contrast to SIR behavior. Note however that there are a small number of messages in which ⟨*k*_⟩ > 2. Perhaps this indicates that while most of the highly popular messages will follow the FSIR mechanism below the critical threshold, there are some that are above it. These do not tend to be among the most widely spread, however, and this could be due to their overall short lifespan. For example, a message about an upcoming election will be of interest to users prior to the election day. After the election the message disappears.

## The phase transition of the FSIR model

**Fig 4:**
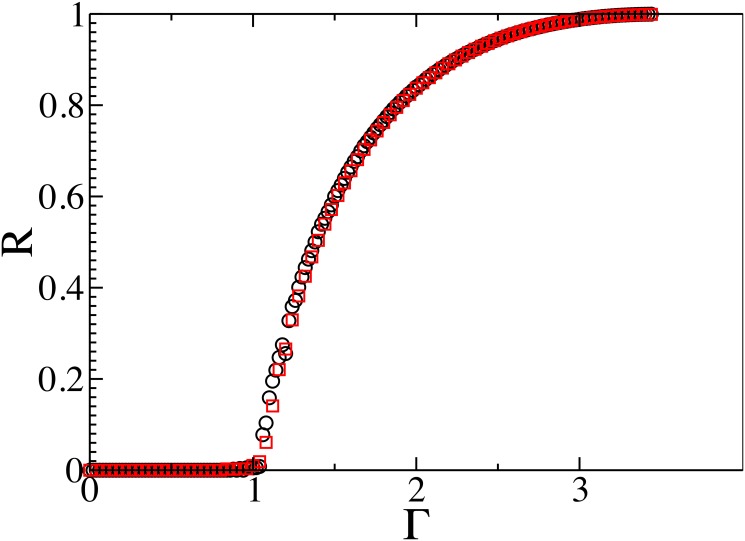
*R* as a function of Γ for τ = 2 with *N* = 10^5^ on SF networks with λ = 2.5 (black) and λ = 3 (red) and *k*_*min*_ = 20. As shown in the plots FSIR has a critical point around Γ_*c*_ ≈ 1, independent of the degree distribution. The same critical value Γ_*c*_ is observed for other degree distributions with different parameter settings.

We next analyze the impact of the FSIR mechanism on information epidemics. On scale-free networks (or networks with broad degree distributions) the SIR mechanism has a critical threshold for epidemics of Γ_*c*_ ≈ 0. This means that any disease is able to infect a considerable proportion of a population regardless of its intrinsic ability to spread. As suggested above, the spread of information in a OSN follows the FSIR mechanism, and thus, as we will show below, the critical threshold is Γ_*c*_ ≈ 1. Below this critical Γ_*c*_ threshold, information “outbreaks” can develop but not information “epidemics”. Above this threshold the information can diffuse to a much wider finite fraction of the user population. Fig 4 plots the FSIR simulation results for scale-free networks with two different values of λ. In these simulations *N* = 100,000, τ = 2, and *k*_min_ = 20 for λ = 2.5 and λ = 3. As Γ increases the FSIR shows a critical phase transition at Γ_*c*_ ≈ 1, which differs from the SIR [12] in which Γ_*c*_ ≈ 0 for *N* → ∞.

For a random network with degree distribution *P*(*k*) in the FSIR model, *T*_*k*_ ≈ Γ/*k*. Using the generating function formalism [10, 41–43] for an inhomogeneous *T*_*k*_, we can derive the probability *f*_∞_ that a branch of infection reaches infinity using the self-consistent equation

$$f_{\infty }=1-G_{1}\left(1-\frac{\Gamma }{k}f_{\infty }\right),$$ (1)

where

$$G_{1}\left(1-\frac{\Gamma }{k}x\right)=\sum_{k}\frac{kP(k)}{\left\langle k\right\rangle }{\left(1-\frac{\Gamma }{k}x\right)}^{k-1}.$$ (2)

**Fig 5:**
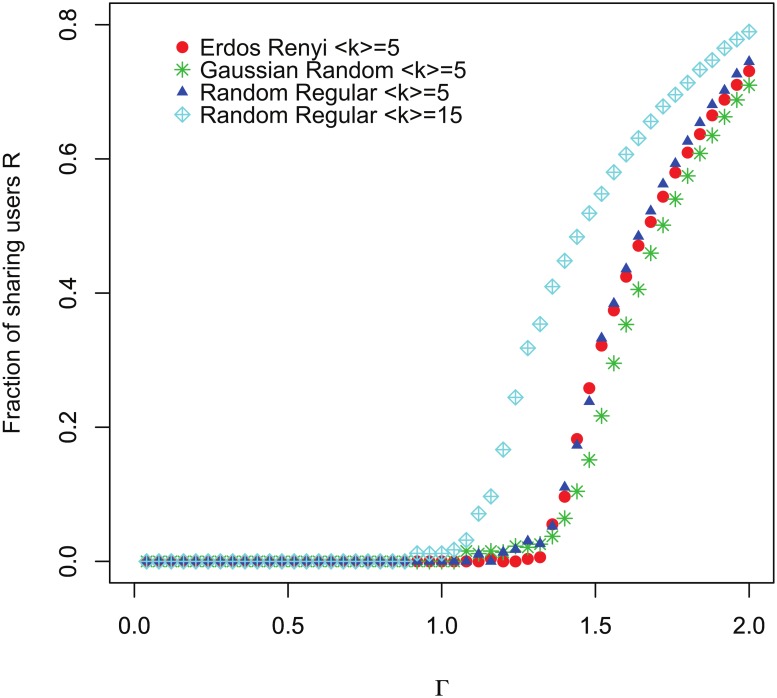
Plot of *R* as a function of Γ for different types of random networks. Regardless of the types of networks, the critical threshold for phase transition Γ_*c*_ is always larger than 1.0. For networks with larger ⟨*k*⟩ values, Γ_*c*_ is closer to 1.0 as predicted by Eq (4).

The non-trivial solution of Eq (1) gives the value of *f*_∞_ for a given value of Γ that corresponds to the intersection between the identity and the right side of Eq (1). At criticality there is only one root that corresponds to *f*_∞_ = 0. Thus the left side of Eq (1) must be tangent to the identity at *f*_∞_ = 0. In other words, the derivative of Eq (1) evaluated on this root must be one. Thus

$$\Gamma_{c}\;\frac{\sum_{k}\left(k-1\right)P\left(k\right)}{\left\langle k\right\rangle }=1,$$ (3)

and

$$\Gamma_{c}=\frac{\left\langle k\right\rangle }{\langle k\rangle -1},$$ (4)

independent of the degree distribution *P*(*k*) (see Fig 5). In a real OSN in which ⟨*k*⟩ is of the order of 100, Γ_*c*_ ≈ 1.

The fact that Γ_*c*_ = 1 has a significant impact on information epidemics means that, in an OSN, most messages cause small information outbreaks at best, and only highly attractive and interesting messages (Γ > 1) reach a significant fraction *R* of the user population. The Γ value of individual messages depends not only on how attractive, interesting, or novel a particular message is, but also on how attractive, interesting, or novel all the other messages being shared during the same period are. The novelty and attractiveness of all messages can theoretically double, i.e., the Γ value of every message can double, but the attention of the users cannot. Thus what matters is the Γ value of a particular message as compared to the Γ values of all the other messages, because all messages are competing for attention from the same user population. In other words, γ values can indicate the weighted novelty of a message compared to all other messages. If there are too many messages with similar a level of novelty, it is probable that none of them will have Γ > 1, and thus none will prevail. When there is an individual message that much more interesting than all the other messages, it may capture a considerable fraction of the user population.

## Discussion

Note that the FSIR mechanism is more likely to be present in situations of information overload. When there is an information overload, users select what they will share and what they will ignore. This is what makes information spreading distinctively different from the spread of disease. A piece of information competes with all other pieces of information for the attention of users (“nodes”), but disease epidemics can utilize every opportunity to spread to other individuals irrespective of the presence of other diseases. In fact, an individual who has been exposed to many diseases is *more likely* to be infected. In contrast, an individual inundated by an overload of messages is *less likely* to view, remember, or pass on any of them.

Information overload also shortens the visibility duration for popular messages. Because messages come to a user every day, new messages appearing above old messages, an information overload means any message, however popular, will rapidly lose its visibility, thus effectively shortening the τ value. Even extremely popular units of information content die quickly. An outstanding example was the immensely popular but short-lived “Gangnam Style” music video that quickly spread across the world—receiving over one billion views on Youtube—and then soon after lost its popularity. There are many other current examples in pop culture and on the Internet of subjects that quickly become world-wide topics and then quickly disappear. In marketing also, many products compete with each other and overload consumers with advertising messages, which may influence them, and their choices may in turn influence their friends, but the result is usually a handful of brands dominating a market with the rest of the brands fighting for survival.

Because this critical phenomenon is present irrespective of network structure, and because people encounter information overload in many settings, the FSIR mechanism and its associated phase transition phenomenon are present in many other real-world contexts. As information spreads across an OSN according to either the SIR or FSIR mechanism, any attempt to predict the popularity of a unit of information content must first determine which mechanism is present. Only then can business or government institutions, for example, obtain useful insights into the behavior of information speading.

The bigger question raised from this work is what determines the spreading mechanism of a message, and what contributes to its novelty value (Γ value). Related empirical studies [44, 45] on social contagion of products provided valuable insights into some important features of marketing campaigns that induces viral spreading. Our work provides a systematic framework to mathematically relate such features to adoption rates. We believe that, combining our mathematical framework with large pool of data and the right methodology from machine learning and text mining, more can be achieved in this field with immense social values.

## Supporting Information

S1 File Microblog spreading data. This zip file contains the sharing details of all of the microblogs we have used in the Figs 2 and 3. Among the files, ‘Instruction.txt’ describes the contents of the files.
(ZIP)
